# An Unfortunate Criticism and a Threatened Boycott

**Published:** 1888-08

**Authors:** 


### AN UNFORTUNATE CRITICISM AND A THREATENED BOYCOTT.

The editorial staff of the New Orleans Medical and Surgical Journal is composed of seven physicians and a lay man; the physicians all being members of the Louisiana State Medical Society. It seems that none of them attended the recent meeting of the society at Monroe, notwithstanding several of them, we understand, hold important positions,—one being chairman of committee on "revision of Constitution," a movement set on foot at his suggestion. Notwithstanding this fact, and judging from the report of the proceedings, our distinguished contemporary published a most scathing, and we think, unjust criticism, not only of the meeting, but of the society, characterizing the meeting as made up of a "few straggling, languid, inconsequent men" (or physicians—we quote from memory) "assembled for the most part for a few days of cigar smoking and story-telling;" and denouncing the proceedings as "slipshod and indifferent," expressing the opinion that the society should be dissolved as "a useless consumer of time and an encumberer of the earth"! !

This naturally fired the indignation of the members—some two hundred of the best known physicians in the State, amongst them the venerable and distinguished Drs. R. H. Day and Joseph Jones, ex-Presidents; the latter, the able and distinguished President of Louisiana State Board of Health, and they are out in a circular, denouncing *as false*, the statements made in said editorial; and in another circular signed by Dr. Fox, of Jesuits Bend, ex-President of the society, and a long list of other prominent members, they recommend the expulsion from the society of the editors of the Journal, and also from the American Medical Association, if any are members, and advise the physicians of the State, to withdraw their support from the Journal.

This would be a serious, if not fatal blow to that able organ, and we sincerely trust that some measure of reconciliation and adjustment may yet be devised whereby the boycott may be avoided. The signers of the circular suggest the founding of an organ of the society after the manner of the Journal of the American Medical Association.

It is with reluctance that we express an opinion on this unfortunate controversy, but it does seem to us that if failure to make the meetings a success had followed prompt attendance and persistent effort on the part of every member of the staff of the New Orleans Medical and Surgical Journal, (who were in position to materially aid the cause), there would perhaps have been some justification for writing a sharp critisism; but, in our opinion, nothing can justify such a criticism as the one alluded to, and especially, considering the fact that it does not appear that these members did anything to bring about a better state of things; but, on the contrary, neglected important duties assigned them, (some of them), the failure of performance of which must have contributed to weaken the interest of that particular meeting. Certainly, the course of the Journal is iconoclastic, rather than calculated to foster organization, build up the society and advance the interest of medical science in the Pelican State. The worst feature in the unfortunate affair is, that the Journal has been the beneficiary of the society in the matter of receiving for publication not only the reports, but papers read at the sessions. The editorial referred to was as surprising as it was unjust. Surely, the heat of the Crescent City must have affected the brain of our usually cool and level headed confrere.

Come over to Austin ; enjoy the delightful sea breezes and luscious fruits of this favored clime, and cool off.

